# Perspectives, preferences and needs regarding early prediction of preeclampsia in Dutch pregnant women: a qualitative study

**DOI:** 10.1186/s12884-016-1195-2

**Published:** 2017-01-07

**Authors:** Neeltje M. T. H. Crombag, Marije Lamain-de Ruiter, Anneke Kwee, Peter C. J. I. Schielen, Jozien M. Bensing, Gerard H. A. Visser, Arie Franx, Maria P. H. Koster

**Affiliations:** 1 Department of Obstetrics, University Medical Center Utrecht, Room KE04.123.1, P.O. Box 85090, 3508AB Utrecht, The Netherlands; 2 Centre for Infectious Diseases Research, Diagnostics and Screening (IDS), National Institute for Public Health and the Environment (RIVM), Bilthoven, The Netherlands; 3 Faculty of Social and Behavioural Sciences, Utrecht University, Utrecht, The Netherlands; 4 The Netherlands Institute for Health Services Research Utrecht, Utrecht, The Netherlands; 5 Department of obstetrics and gynaecology, Erasmus Medical Center Rotterdam, Rotterdam, The Netherlands

**Keywords:** Preeclampsia, Screening, Attitudes, Preferences, Need, Qualitative research

## Abstract

**Background:**

To improve early risk-identification in pregnancy, research on prediction models for common pregnancy complications is ongoing. Therefore, it was the aim of this study to explore pregnant women’s perceptions, preferences and needs regarding prediction models for first trimester screening for common pregnancy complications, such as preeclampsia, to support future implementation.

**Method:**

Ten focus groups (of which five with primiparous and five with multiparous women) were conducted (*n* = 45). Six focus groups were conducted in urban regions and four in rural regions. All focus group discussions were audio taped and NVIVO was used in order to facilitate the thematic analysis conducted by the researchers.

**Results:**

Women in this study had a positive attitude towards first trimester screening for preeclampsia using prediction models. Reassurance when determined as low-risk was a major need for using the test. Self-monitoring, early recognition and intensive monitoring were considered benefits of using prediction models in case of a high-risk. Women acknowledged that high-risk determination could cause (unnecessary) anxiety, but it was expected that personal and professional interventions would level out this anxiety.

**Conclusion:**

Women in this study had positive attitudes towards preeclampsia screening. Self-monitoring, together with increased alertness of healthcare professionals, would enable them to take active actions to improve pregnancy outcomes. This attitude enhances the opportunities for prevention, early recognition and treatment of preeclampsia and probably other adverse pregnancy outcomes.

## Background

Preeclampsia (PE) is a pregnancy complication characterised by hypertension and proteinuria, sometimes progressing in a multi-organ cluster of varying clinical features [1]. PE complicates 2–9% of all pregnancies and is one of the major causes of maternal and perinatal mortality and morbidity [2].

Risk identification is an essential element of antenatal care and research on its improvement is ongoing. In particular, a large number of first-trimester prediction models for PE have been developed [3, 4]. Applying these prediction models may improve risk selection by early identification and also leaves room for preventive measures, such as the administration of low dose aspirin [5–7]. Prediction models can categorise women into low- and high-risk groups and women will subsequently receive care according to the identified risk (tailored care pathways) [8]. Low-risk women would not need additional surveillance during pregnancy, whereas high-risk women can benefit from supplementation of aspirin and calcium and intensive monitoring (for example Doppler measurements of the uterine arteries, frequent blood pressure checks and/or urine checks for proteinuria).

The potential drawbacks and ethical concerns of the use of such prediction models are related to the false positive results (incorrectly categorising healthy women as at increased risk) and false negative results (incorrectly categorising healthy women as at low-risk). This may lead to unnecessary anxiety and stress, unnecessary prenatal visits and unnecessary prophylactic medication in women who are incorrectly categorised as high-risk [9]. When incorrectly identified as low-risk, this may lead to incorrect feelings of reassurance and delayed identification when the condition occurs. However, these drawbacks are mainly theory driven, as the true perspectives, preferences and needs of pregnant women are unknown. To meet the needs of users of care, it is necessary to develop healthcare that reflects patients’ views and preferences [10–12].

## Methods

The aim of the study was to explore pregnant women’s perceptions, needs and preferences regarding prediction models for preeclampsia and subsequent healthcare pathways, to support future implementation. A focus group approach was used to address the research question. In focus groups data are generated by interaction between participants representing a ‘natural-environment’. Participants present their own views but also hear views and experiences from other participants. By responding to each other they reveal more of their own frame of reference [13].

### Recruitment of participants

As the majority of Dutch pregnant women start their pregnancy in primary care (midwife or general practitioner), the participants for this study were recruited from 11 community midwife practices in the centre of the Netherlands by purposive sampling. The focus groups were conducted between April 2014–July 2015. Potential participants had to be 18 years or older, with a gestational age between 10 and 24 weeks, a singleton pregnancy, and no pregnancy related complications in their current or previous pregnancies. During recruitment gestational age was maximized to 26 weeks in order to conduct a focus group discussion with enough participants.

Women were recruited from midwifery practices. Women who expressed interest in participating in the study received written information, gave schedule opportunities, provided contact details and were assigned to a focus group based on parity, anticipating possible differences between nulliparous and multiparous women.

Utilisation of prenatal screening differs between geographic regions [14–16]. Therefore, we recruited women from high- as well as low-urbanised regions. The degree of urbanisation was determined by the surrounding address density of a neighbourhood, district or municipality. This is a standardized method and represents the average number of addresses per square kilometre within a radius of one kilometre on 1 January of the year 2014 [17]. Midwifery practices located in a region with a surrounding address density more than 2500 addresses per km^2^ were determined as high urbanised, surrounding address density less than 1000–1500 addresses per km^2^ as low urbanised. First, data were collected in high urbanised (urban) regions, next we collected data in low urbanised (rural) regions.

### Data collection

Since prediction models for preeclampsia and subsequent healthcare pathways are not yet integrated in routine prenatal care in the Netherlands, measurement of perceptions, needs and preferences was a challenge. To provide greater focus and specificity to the arguments for an in-depth discussion, we decided on using a disease scenario combined with information on current maternity care and description of future care, presented in a video [18–20]. To ensure that medical facts were accurate, equal, relevant, balanced and neutral, and to ensure that the information was easy to understand for people who are not experts, a group of medical experts and (pregnant) women guided and approved the script for the video. The video provided information on the disease scenario of PE, information on current obstetric care, and a description of future care. After watching the video, the participants received a written summary of the information given in the video. A translation of the summary given to the participants can be found in Appendix.

Before the focus group session, all participants were asked to complete a brief questionnaire to collect information on demographics and a personal history (age, work experience, country of origin, parity and personal and general experience with preeclampsia). All focus groups were performed with a moderator guide. At each session a moderator was present to guide the discussion and a minutes secretary to take notes. “One researcher (MLR) and two research-assistants (MC & JdB), with a background in moderating focus groups, acted as moderators. They were fully instructed on the research- and interview protocol. The role of the moderator was to guide the discussion and listen to what is said, and not to participate, share views, engage in the discussion or shape the outcome of the group interview. The moderator summarised the discussion and after each session debriefs with the minutes secretary. The sessions were audio and video-taped and viewed after each session by one researcher who had not participated or moderated the focus group interviews (NC) [21]. The focus group sessions were conducted using a semi-structured interview protocol which comprised the following topics: participants’ knowledge of the target condition (i.e. PE); participants experience and perception towards PE; preferences and need regarding utilisation of prediction models for PE; advantages and disadvantages of prediction models for PE; level and amount of pre- and post-test counselling and information. Focus group sessions lasted approximately 90 minutes, were digitally recorded (audio and video) and transcribed verbatim. After six focus groups in urban regions and four in rural regions, no new information was collected anymore and it was agreed that saturation was reached and recruitment was stopped. Thus, we performed 10 focus groups comprising a total of 45 pregnant participants.

### Data analysis

Data analysis was performed on basic content, to describe and categorize arguments and to identify links between women’s characteristics and arguments. The transcripts were coded systematically to identify patterns and major themes in the focus group transcripts. To capture the group dynamics and interactions between individual participants we have used whole group analysis as described by Spencer et al. [22]. Data produced by the group are used as a whole without specifying individual contributions. The group will then become the unit of analysis and will be treated as unit of individual data.

**Fig. 1:**
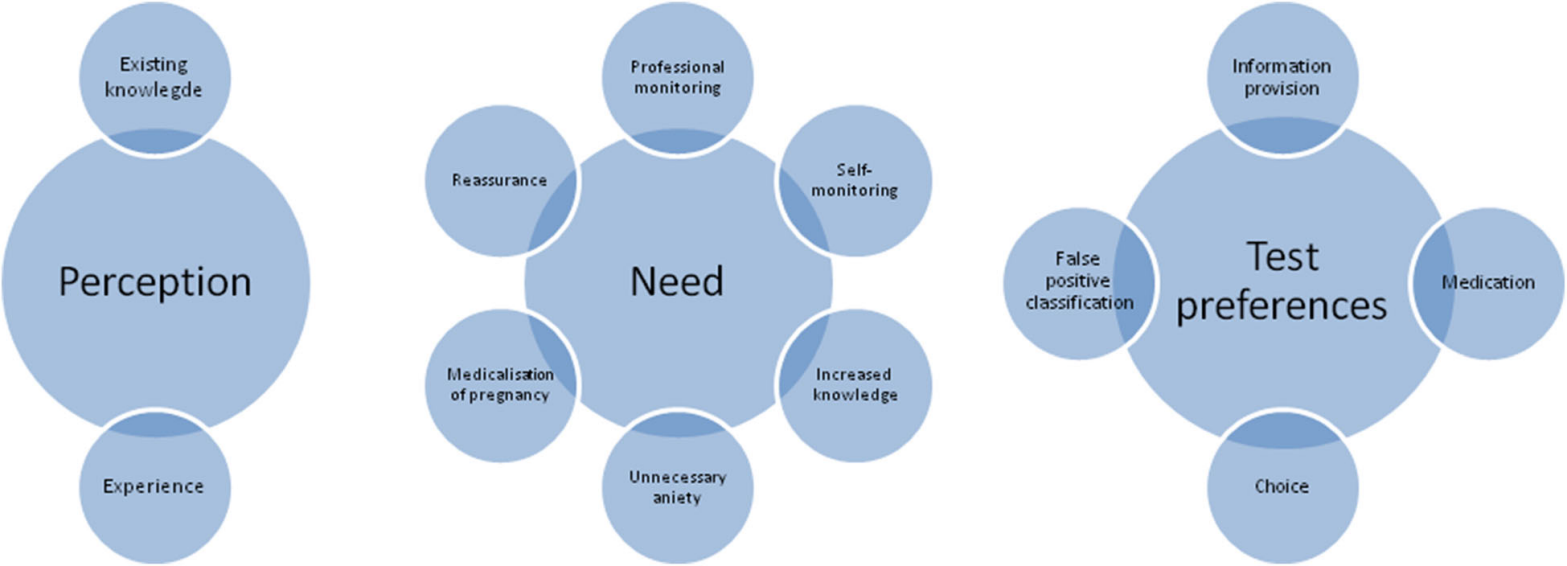
Categories and subcategories of coded themes used in this study

The focus group transcripts were systematically coded (thematic analysis) by using the computer software Nvivo10. First, we conducted open coding, in which we assigned initial codes to text fragments. Two researchers (NC and DM or TK) independently conducted analysis of the transcript to reach an understanding on the assigned open codes (subcategories). Next, the initial codes were combined by making connections between categories and placing them in a broader context related to the research subject [23]. This resulted in three described and arranged categories: perceptions, needs, and preferences. For example, the coding themes ‘reassurance’, ‘professional monitoring’, ‘self-monitoring’ and ‘increased knowledge’ all became subcategories of ‘need (for testing)’ (Fig. 1).

## Results

In general, the focus groups were lively and participants were motivated to participate. The women participating in the focus groups were able to formulate arguments and the group interaction contributed to deepen the discussions.

### Baseline characteristics

A total of 10 focus groups were conducted (*n* = 45). Six focus groups with women residing in highly urbanised regions (*n* = 27), four focus groups with women residing in low urbanised regions (*n* = 18). Within the two regions, half of the groups consisted of primiparous women, half multiparous women. The majority of participants were highly educated, had a paid job and were Dutch. In high-urbanised regions the mean age was 33.7 and in rural regions mean age was 29.6. Participant characteristics are summarized in Table 1.

**Table 1:** Socio-demographic characteristics of the study population in high urbanisation and low urbanisation regions

	High urbanisation (*n* = 27)		Low urbanisation (*n* = 18)	
	Nulliparous (*n* = 11)	Multiparous (*n* = 16)	Nulliparous (*n* = 8)	Multiparous (*n* = 10)
Mean maternal age	33.8	33.7	28.3	30.5
Marital status				
Partner	11	16	8	10
Single	–	–	–	–
Highest education^a^				
Low	–	–	1	–
Intermediate	1	3	3	4
High	10	13	4	6
Occupation				
Paid job	11	16	7	9
Unemployed	–	–	1	–
Housewife	–	–	–	1
Etnic origin^b^				
Dutch	9	14	8	10
Non-Dutch	2	2	–	–
General experience with preeclampsia^c^				
Yes	4	13	5	5
No	7	3	3	5

^a^Education was defined as ‘low’ (elementary school, lower level of secondary school), ‘Intermediate’ (higher level of secondary school and intermediate vocational training) and ‘high’ (higher vocational training and university)

^*b*^Ethnic origin in the Netherlands is defined by country of birth of a person’s parents. If one or both parents are born outside the Netherlands a person is considered non-Dutch (Dutch National Office of Statistics; Statistics of the Netherlands)

^c^General experience was described as ‘have or have not people in your social environment who have experienced preeclampsia’

### Perception

Most women had some knowledge of PE, mainly based on experiences from friends, family or colleagues. These women considered PE a serious complication for both the mother and child (*perception*). All women in this study, except for one who initially had some reservations, had positive attitudes towards prediction models for PE. When available, all women would eventually participate.

### Needs

Arguments shared in the focus groups were mainly in favour of prediction models for PE. In general, women preferred to receive more information on possible complications in pregnancy. Some multiparous women even regretted not being informed about possible complications in an earlier pregnancy to create more alertness (*increased knowledge*).

*‘It worries me that I have not been informed on this (preeclampsia) in my former pregnancy or in this pregnancy […], it is quite a serious condition, and I would have appreciated to receive a list with symptoms to be aware of, just suppose I would have had these symptoms and would not have recognised it. So I am actually more worried about the lack of information’ [F16, multiparous, highly urbanised region]*.

Testing was considered as an additional tool for increased monitoring of their pregnancy, both by themselves and their healthcare providers/midwives. Knowledge on their personal (high) risk was perceived beneficial as this would enable them to pay more specific attention to signals potentially related to PE (defined as self-monitoring), while they also expected their healthcare professionals to be more alert on specific symptoms. As a result they expected improvement in early recognition of complications and timely consultation of a healthcare provider. Knowledge on their personal (high) risk would also enable women to take preventive actions if possible (*professional- and self-monitoring*).

*‘If you would receive information like “be aware of headache and blurred vision” and the information like how it was presented in the information video, that will possibly increase your alertness’ [P6, primiparous, low urbanised region]*.

*‘And suppose PE screening will be implemented, they (healthcare professionals) will be aware of your high-risk, and consequently be more alert, when compared to being at low-risk’ [M9, multiparous, low urbanised region]*.

Anxiety and reassurance played a major role in the need to be screened. Being categorised as low-risk was considered reassuring and was for some women the main reason they would opt for screening, although they acknowledged that the result was a risk estimation only (*reassurance)*. At the same time, it was acknowledged that a high-risk result could cause increased anxiety, but possibilities of self-monitoring, preventive means and more intensive professional monitoring could possibly level out these feelings of anxiety (*anxiety*).

*‘….also because there will be more consultations, there will be more often a moment of reassurance. And if the healthcare professional pays close attention to my condition, this will possibly compensate my feelings of anxiety due to a high-risk” [F4, primiparous, highly urbanised region]*.

Few women had doubts regarding participation in screening for PE. These women felt that screening may cause unnecessary anxiety and unnecessary medicalisation of pregnancy. They expected that prior to severe pregnancy complications, they would experience alarming physical complaints, so feeling in good shape was reassuring enough (*medicalisation*).

*‘On the other hand, screening could also cause unnecessary anxiety, as women will be worried and think: “O no, I have a high-risk, so I will get this disease” [F22, multiparous, highly urbanised region]*.

*‘..I find it important to be screened, but you also have to take things as they come. It is your body doing the work and you have to trust on the signals of your body’ [M1, multiparous, low urbanised region]*.

### Preferences

The participants in this study gave clear views on preferences they had regarding a hypothetical test-offer. Quotes related to test preferences are given in Table 2. Prior to screening they would like to be informed on the screening method in general. They also wished to be informed about the condition and how screening results would be beneficial. Moreover, women would like information on the consequences and follow-up in case of a high-risk. Information should be provided by their midwife at the first consultation. Leaflets, specific websites or information meetings prior to screening were appreciated to support oral information. Results should be communicated in person, preferably with an option to discuss a screen positive result. Women agreed that post-counselling information, in case of a high-risk, was essential. Information provision in case of low-risk results should be focused on symptoms to create general awareness (*Information provision*).

**Table 2:** Study participants test-preferences regarding the hypothetical offer of prediction models for preeclampsia

Information provision	*‘I would like to know these percentages, only information ‘you are categorised as high or low-risk’ for me is not enough, I want complete information. For example what does it mean to be at low-risk, and what are my chances of developing the condition. And what does it mean to be at high-risk and what are my chances of developing the condition then. What are options for prevention and treatment. And when I know all these things, what are my options? [F9, primiparous, highly urbanised region].*
	*‘Some oral information, but also some written information, so you can look the information up when you are back home’. [P8, primiparous, low urbanised region]*
Medication	*‘I am positive towards early identification of your risk, but I feel some reluctance for taking aspirin throughout my pregnancy. Calcium, for me is OK, you can find this in pregnancy vitamins as well’ [P8, primiparous, low urbanised region].*
	*‘I found it a bit strange, I have no medical background, but I have been told that in case of a headache or any pain, it is better not to take aspirin as a painkiller, but paracetamol instead’ [F15 multiparous, highly urbanised region].*
Choice	*I think that everybody should make their own choices (to participate or not). That is my opinion [P7, primiparous, low urbanised region]*.
	*‘I would like to hear this (screening for PE) afterwards. They will take a blood test anyway, and then they will do all these exams....I don’t mind. And if they inform me about this afterwards, that is early enough’ [F1, primiparous, highly urbanised region].*
	*‘Regarding use of aspirin and calcium, for me this should be a choice. First I would like to receive extensive information about these medicines [….] and than discuss what are the options [F4 primiparous, highly urbanised region].*
False-positive classification	*‘I fully agree…for me it would be really reassuring, because if you are determined as high-risk, there is only a 20% chance of developing the disease. Well, if I would have a chance of 80% to win the lottery, I would certainly participate. I don’t think it (high-risk identification) would worry me’ [F13, multiparous, highly urbanised region].*

Opinions were divided regarding the use of preventive medication. In particular, women did not like the idea of using medication in case of a false positive screening result. Calcium supplements were considered less problematic as calcium was considered as a natural substance, whereas aspirin was perceived as a drug. Explicit information on the effects of medication, long-term side effects and proven effectiveness were considered crucial for the use of preventive medication *(Medication)*.

After receiving information prior to screening (counselling) most women preferred to have a choice whether or not to be screened, although for some women this pre-test counselling was not necessary. The majority of women also wanted to have a choice whether or not to take preventive medication. Some also preferred to have a choice in who would be responsible for the follow-up after a high-risk result (*Choice*).

The possibility of receiving a false positive result was not perceived as something negative. Women found it reassuring to know that in case of determined high-risk, the chances of developing the condition are small. Besides, a high-risk would give them direct access to specialised care, which was a reassuring thought for most women (*False positive results*).

### Subgroup analysis

Multiparous women more often referred to earlier experiences, which mostly provided them with more confidence and knowledge. Therefore, for some, referral to specialised care in case of high-risk was not essential. They suggested a combined pathway (midwife/obstetrician) or intensive pathway with a midwife only (*primary care*).

*‘A gynaecologist is in a hospital, with different smell, different light....A midwife more often is close to your house, whereas a gynaecologist is not. A midwife is easy accessible, but if you have a high-risk.....well, I don’t think it is necessary to be with a gynaecologist for the rest of your pregnancy’ [M3, multiparous, low urbanised region]*.

Nulliparous women needed more support and confirmation of their wellbeing. In this group there was a more positive attitude towards specialised care, in which they expected to receive more intensive monitoring, which would give them more reassurance (*specialised care*).

*‘I would prefer to have direct access to specialised care in case of any alarming symptoms or feelings of discomfort, instead of convincing someone that they have to take my complaints in serious consideration. So yes, from that point of view, being at high-risk provides me with this direct access*’ [*F5, primiparous, highly urbanised region]*.

Women in highly urbanised regions more often felt the need to receive as much information as possible and intensive monitoring of their pregnancy. They more often felt that specialised care was more reassuring and did not mind to go to the hospital to receive care. Women from low-urbanised regions also felt the need to receive information, but more often preferred care close to their homes (mostly being midwifery care). Besides, they appreciated the personal care and easy access and felt confident that their midwife was well equipped to monitor their health. They more often referred to ‘trust in their own bodies’ and ‘not to create problems if not present’.

## Discussion

### Main findings

The purpose of this study was to explore perceptions, needs and preferences of pregnant women regarding prediction models for preeclampsia and subsequent care. The results of our study suggest that the majority of women in this study had a positive attitude towards preeclampsia screening and were willing to participate when available, which is in line with earlier research regarding PE screening [24, 25]. When identified as high-risk, self-monitoring, together with increased alertness of healthcare professionals, was perceived as a means to take active actions to potentially improve pregnancy outcomes. Whether self-monitoring is an effective screening instrument for PE is not clear and should be further studied, but for our study participants it was experienced as something positive as they felt they could play a role in early detection or even play an active role in care for their pregnancy themselves.

In the Netherlands pregnant women are initially considered as low-risk, and the majority start their pregnancy in primary care [26], characterised by its low-medicalised approach and prevention of unnecessary concerns and anxiety [27]. Anxiety in the perinatal period is common among pregnant women and increases throughout pregnancy [28, 29]. Participants in our study also anticipated the possibility of elevated feelings of anxiety. But by active engagement and alertness in their personal health, they expected the disadvantage of anxiety to be levelled out. Which is in line with findings of Simeone et al. [25], who demonstrated that participating in a first-trimester preventive program for PE did not increase levels of anxiety [25].

In this study the majority of women had no problem with specialised care in case they should be classified as high-risk, while some even preferred specialised care in general. This is an interesting finding, as the Dutch obstetric system in which these women received care is midwifery-led and characterised by its low-medicalised approach and high satisfaction rates are suggested with this type of care [30–32].

Preference for specialised care was more (but not solely) present in women living in the urban region and in nulliparous women. A possible explanation for this is that for women residing in a city, accessibility to a hospital or midwifery practice is equal, whereas for women in rural areas this is not. Nulliparous women lack the experience of a prior pregnancy and might experience more feelings of uncertainty. Increased interventions and monitoring possibly gives them a feeling of control over the situation and therefore more reassurance.

### Strengths and limitations

This study highlights the preferences, perceptions and needs of the care-users, and provides important information to understand women’s perspective. Incorporating the patient perspective is important for the development of patient centred care.

The results of this study were mainly in favour of screening for maternal complications, but some weaknesses of our study need to be addressed. Numbers of participants were low and most were highly educated and were Dutch. The participants’ characteristics, such as educational level, may have biased our results. Therefore future research should focus on generalisation of our findings and should be tested in large representative groups of women. Furthermore, one multiparous woman was erroneously allocated to a focus group of nulliparous women and may have influenced the nulliparous women’s opinions. One group of multiparous women included a woman that had had PE in a former pregnancy and that may have influenced the other participants. Nevertheless, the findings in both groups were not substantially different from the other groups.

### Interpretation

In general, people have a preference to have influence on events in their life [33]. By knowing a possible high-risk, women may feel they can take action to improve their outcomes. This is in line with earlier findings regarding women provided with risk information for preeclampsia. These women were very receptive to more intensive monitoring and engaged in efforts to reduce their risk of preeclampsia, a phenomenon also known as the illusion of control [24]. Certain activities, such as active involvement, gives people the feeling that they have more control over a situation [34]. So, if a test provides information on someone’s (near) future, if they have the feeling they can improve the situation, people are more willing to take a test, which is in line with findings regarding genetic testing [35].

The findings of our study potentially open up possibilities for the use of PE screening in current obstetric care [24]. There is some evidence on preventive effects of low-dose aspirin and calcium [36] and early risk identification can support healthcare professionals and prospective mothers in early recognition of the onset of the disease. This might be an important improvement, as severe complications in PE are related to substandard care and relatively late identification of the disease [37, 38]. Perhaps the most important potential benefit of PE screening is increased cost-effectiveness. It allows intensified surveillance in women identified as at high-risk and avoidance of too many antenatal visits, diagnostic and therapeutic interventions in those identified as at low-risk.

On the contrary, as there is still no real cure for PE, we should also be aware of not giving women the illusion of reassurance by the increase of medical interventions. Therefore, implementation of PE testing can be of added value, but should be carefully implemented. The results of our study provided us with some meaningful suggestions from the study participants regarding future implementation of the test. Obviously, they were related to the personal needs of these participants, but they also reflected on possible drawbacks. As a result, we suggest that prediction models for PE should be offered with clear and realistic oral pre-test information in which the pregnant woman is a partner on equal term. Unsolicited support when at high-risk to reduce stress and support coping [39] was among the suggestions. The use of medication should be well supported in terms of safety, reliability, usefulness and long-term effects.

The positive attitude towards PE screening as found in this study differs greatly from that on Down syndrome screening in the Netherlands. The latter only has an uptake of 27% [40]. In the present focus groups we did not address this issue, but differences in attitude may relate to ethical considerations related to the treatment options and feelings of anxiety [14, 41]. Early studies suggest that anxiety levels are more elevated following a screening test that has impact on foetal health, than following one that has impact on maternal health [42]. Being able to observe one selves more intense, apply preventive means and receive more intense care are seen as relevant interventions to be prepared or even improve pregnancy outcomes.

## Conclusion

In conclusion, women in this study had positive attitudes towards PE screening. Identification of women at high-risk offers opportunities for prevention, early recognition and treatment. Because of the lack of proven effective prevention of PE and the possibility of misclassification pre and post test counselling remains essential.

## Appendix

Translation of summary provided to the focus group participants.

Advantages versus disadvantages of current risk stratification strategy in the Netherlands.

**Table:**

Advantages	Disadvantages
• Pregnant women will only be classified as ‘ill’ if the condition (preeclampsia) is indeed diagnosed	• The onset of preeclampsia can be suddenly and severe, the condition can only be diagnosed when the pregnant women is already (severly) ill
• No unnecessary concern will be raised in the beginning of pregnancy	• Only women who had a previous preeclampsia will receive intensive monitoring during pregnancy
• No medical interventions unless the condition is diagnosed	

Low risk care pathway – guided by prediction model.

- Prenatal visits in primary care (midwife).
- Similar to current prenatal care.

High risk care pathway – guided by prediction model.

- Prenatal visits in secondary care.
- Supplementation of low-dose aspirin and calcium.
- Doppler measurement of the uterine arteries at 20 weeks anatomy scan.
- Follow-up of fetal growth by ultrasound (not routinely performed in the Netherlands).
- Check for proteinuria.

Advantages versus disadvantages of proposed risk stratification strategy in the Netherlands.

**Table:**

Advantages	Disadvantages
• Pregnant women who are considered low-risk truly have a low risk of developing preeclampsia	• 80% of women who are considered high-risk will receive intensive care while they would never have developed preeclampsia
• Pregnant women who are considered high-risk can lower this risk by supplementation of low-dose aspirin and calcium	• These women may experience unnecessary anxiety
• Using prediction models for preeclampsia allows for personalized tailored care	• 1% of women considered low-risk will still develop preeclampsia

## Abbreviation

PE: Preeclamsia
